# Troubling Neurobiological Vulnerability: Psychiatric Risk and the Adverse Milieu in Environmental Epigenetics Research

**DOI:** 10.3389/fsoc.2021.635986

**Published:** 2021-04-12

**Authors:** Angela Marques Filipe, Stephanie Lloyd, Alexandre Larivée

**Affiliations:** ^1^Department of Sociology and Centre for Research on Children & Families, McGill University, Montréal, QC, Canada; ^2^Centre for Biomedicine, Self & Society, Usher Institute, University of Edinburgh, Edinburgh, United Kingdom; ^3^Department of Anthropology, Université Laval, Québec, QC, Canada

**Keywords:** early life adversity, vulnerability, risk, epigenetics, neuropsychiatry, milieu, Canguilhem, ecosocial theory

## Abstract

In post-genomic science, the development of etiological models of neurobiological vulnerability to psychiatric risk has expanded exponentially in recent decades, particularly since the neuromolecular and biosocial turns in basic research. Among this research is that of McGill Group for Suicide Studies (MGSS) whose work centers on the identification of major risk factors and epigenetic traits that help to identify a specific profile of vulnerability to psychiatric conditions (e.g., depression) and predict high-risk behaviors (e.g., suicidality). Although the MGSS has attracted attention for its environmental epigenetic models of suicide risk over the years and the translation of findings from rodent studies into human populations, its overall agenda includes multiple research axes, ranging from retrospective studies to clinical and epidemiological research. Common to these research axes is a concern with the long-term effects of adverse experiences on maladaptive trajectories and negative mental health outcomes. As these findings converge with post-genomic understandings of health and also translate into new orientations in global public health, our article queries the ways in which neurobiological vulnerability is traced, measured, and profiled in environmental epigenetics and in the MGSS research. Inspired by the philosophy of Georges Canguilhem and by literature from the social studies of risk and critical public health, we explore how the epigenetic models of neurobiological vulnerability tie into a particular way of thinking about the normal, the pathological, and the milieu in terms of risk. Through this exploration, we examine how early life adversity (ELA) and neurobiological vulnerability are localized and materialized in those emerging models while also considering their broader conceptual and translational implications in the contexts of mental health and global public health interventions. In particular, we consider how narratives of maladaptive trajectories and vulnerable selves who are at risk of harm might stand in as a “new pathological” with healthy trajectories and resilient selves being potentially equated with a “new normal” way of living in the face of adversity. By troubling neurobiological vulnerability as a universal biosocial condition, we suggest that an ecosocial perspective may help us to think differently about the dynamics of mental health and distress in the adverse milieu.

*To act, it is necessary at least to localize. (.) The impetus behind every ontological theory of disease undoubtedly derives from therapeutic need. When we see in every sick [person] someone whose being has been augmented or diminished, we are somewhat reassured, for what [one] has lost can be restored, and what has entered can also leave. (Canguilhem*, *1978**, p. 11)*

## Introduction

Since the start of the twenty-first century, the life sciences have been ushered into a field of research on gene (de)regulation that marked the rise of a post-genomic era in the life sciences. The next few years saw the publication of a highly influential research in which scientists from McGill University correlated early experiences of stress in rodent animal models (i.e., experiences of maternal neglect as opposed to maternal care) with specific patterns of DNA methylation (an epigenetic trait) in offspring (Weaver et al., 2004; Meaney and Szyf, 2005). These initial findings were subsequently translated into studies of human populations by researchers at the McGill Group for Suicide Studies (MGSS), who identified similar molecular biomarkers in human populations and who developed epigenetics' models of the effects of early stress and childhood maltreatment on neurobiological risk trajectories (McGowan et al., 2009; Lutz and Turecki, 2014). Epigenetics research has also furthered our understanding of how life's experiences and living environments may be impressed on the human (epi)genome in ways that affect its expression over time (e.g., by adding and/or removing molecular tags), thereby modulating health and disease outcomes across the lifespan (Niewöhner, 2011; Guthman and Mansfield, 2013; Lappé and Landecker, 2015)^1^. As these concepts of experience and environment came back to the fore in post-genomic, biosocial science, increasing research attention has been paid to the moments at which people are considered to be most susceptible to exposure, which is a central question both in the neuroscience of childhood brain plasticity and in epigenetics studies of vulnerability (Champagne, 2010; McEwen and Morrison, 2013).

Drawing on the philosophy of Georges Canguilhem, the social studies of risk, and critical and global public health literature, we explore how neurobiological vulnerability to psychiatric risk is modeled in environmental epigenetics and asks what the conceptual and translational implications of this research are for how we might conceive of and seek to intervene in mental health. In a context in which researchers seek to explain why some people would respond more negatively than others to similar, even if extreme, exposures to stress, the source of vulnerability has been consistently sought in early life adversity (or ELA, for a sociological debate, see Gillies et al., 2016; Lappé, 2018; Lloyd and Larivée, 2021a)^2^. Among this research is the work of the MGSS on environmental epigenetics models of psychiatric risk, in which adverse experiences in early life are thought to engender vulnerability to a variety of mental health conditions (e.g., depression) and risk behaviors (e.g., suicidality) (Lutz et al., 2017). Of particular concern to the MGSS is the possibility of biological embedment of negative exposures and extreme environments (e.g., childhood abuse) in the form of “maladaptive traits” (Brezo et al., 2008, p. 134) and other epigenetic traits (Lutz et al., 2017; Tanti et al., 2019), which may subsequently manifest in maladaptive trajectories marked by detrimental decision-making, interpersonal, and emotional difficulties, and negative mental health outcomes (Jollant et al., 2010, 2011)^3^. While this research seeks explanations for psychiatric and suicide risk as the extreme end point of a pathological trajectory, the MGSS is more broadly interested in tracing risk factors and localizing neuromolecular marks that help identify specific profiles and predict trajectories of neurobiological vulnerability.

Alongside the work of the MGSS, a burgeoning field of biosocial research has developed at the junction of clinical studies, environmental epigenetics, and longitudinal cohort research with the aim of identifying specific social and physical “exposures” in early life that lead to a range of negative health outcomes later in life (Perng et al., 2019). This research has further converged with global health agendas, such as the Developmental Origins of Health and Disease frameworks (DOHaDs), that are oriented toward the study of developmental pathways of health and illness at the level of the population and toward the mitigation of risk factors for healthier life trajectories (Penkler et al., 2018; Pentecost and Meloni, 2020). The potential translation of epigenetic research into global public health orientations (Rozek et al., 2014) calls for a closer examination of the ways in which ELA-based “vulnerability” is traced, measured, and profiled in epigenetic science. Based on the case study of the MGSS and on an analysis of their scientific output and of recent epigenetics literature, we critically examine how neurobiological vulnerability to psychiatric conditions and suicide risk is construed and modeled in environmental epigenetics and in the wider biosocial research agenda. While this article draws on a critical analysis of scientific research literature, it is more broadly informed by insights from an ongoing, multi-year study of the MGSS that includes interviews with researchers and study participants, as well as laboratory and meeting observations.

The aim of our article is two-fold. First, we examine the materialization of neurobiological vulnerability as an individual susceptibility (to dying by suicide) that can be localized in the brain and a condition (of “living at-risk”) that can be traced back to one's early years. Drawing on the work of Georges Canguilhem, we argue that the model of neurobiological vulnerability that is emerging from environmental epigenetics research ties into a seemingly novel yet persistent way of thinking about the normal, the pathological, and the milieu in terms of risk. Second, we query the environmental epigenetics model of neurobiological vulnerability, whose etiology is attributed to the embedding of ELA and the maladaptive trajectories and responses to the living environment that it ensues. Returning to the opening quote by Georges Canguilhem on the scientific impetus for an ontological theory of disease, we suggest that the epigenetic model of human–environment interactions is characterized by an aspiration to trace the imprinting of stressors on the plastic brain, to localize biomarkers and susceptibility traits, and to potentially intervene in maladaptive trajectories of neuropsychiatric and suicide risk. This epigenetic model implies a particular view of suicide vulnerability and behavior (and their correlated profiles and trajectories of risk), which is built upon currently limited windows into a person's life (e.g., post-mortems on brain tissues and proxy interview data) as we explore in the following sections. Thus, although this model's conceptual architecture builds on the premise that social environments dynamically shape our bodies and brains, its materialization and operationalization rely, to date, on a mechanistic rendering of such dynamics (e.g., on correlations between stress exposures and epigenetic traits and between these and risk factors). More broadly, this model implies a biosocial framing of ELA that is gradually shifting attention from normal and pathological behaviors toward that of vulnerable brains and maladaptive life trajectories as opposed to an ecosocial framing, which considers the full range of interplay between mental health and the environment (or milieu) one inhabits.

We take the idea of the living environment one step further and argue that health and life are always dynamic and situated processes that are shaped by the adversities and contingencies in indeterminate ways (Canguilhem, 2008[1952]). When construed in terms of exposure and adverse experiences, this milieu is seen as the source of risk, vulnerability, and susceptibility to harm, yet it is also one's living milieu that may act as the locus for adaptation, transformation, and repair, the latter being hinted at in the quote by Canguilhem that opens this article. In this vein, we argue that vulnerability might be seen not only as a universal neurobiological (or biosocial) condition of susceptibility to harm or a negative state of fragility lodged within the individual; rather, it may be also seen as a dynamic and contextually heterogeneous aspect of the life of a “person-in-environment.” Turned on its head, vulnerability may be understood as sensitivity and openness to the living milieu and, thus, a harbinger of both negative and positive potentials (c.f., Taussig et al., 2013). After all, risk and resilience, adaptation and vulnerability, health, and illness are construed as dynamic processes that are in interplay with/in the environment—-in both Canguilhem's philosophy and in environmental epigenetics. The next years will be decisive in terms of observing the extent to which epigenetics might be able, by building on early findings and emerging tools, to allow insights into the dynamic relationships between the living and the environment and how they affect the brain, human development, and mental health. By troubling the biosocial model of neurobiological vulnerability to psychiatric illness and suicide risk, we hope to make a first step toward thinking more creatively about social ecologies of vulnerability and the ways in which the living milieu may foster mental health and mental distress.

## Neurobiological Vulnerability: From the Normal and the Pathological to Healthy Lives and Maladaptive Trajectories

Vulnerability is a key word in a burgeoning set of research agendas in environmental epigenetics and developmental neuroscience that are concerned with how specific socio-material exposures and milieux (Boivin et al., 2012; McEwen and Morrison, 2013) may put people at greater or lesser risk of specific developmental and health outcomes. Crucial to the MGSS model of neurobiological vulnerability is the idea that the development of pathological, though potentially reversible, brain-based structural and functional traits (Lutz et al., 2017) is seen as an adaptation to adverse circumstances and, specifically, to ELA (Turecki, 2016; Brown et al., 2019). The model proposed by the MGSS is thus consistent with developmental neuroscientific narratives of early development and brain plasticity as a singularly potent milieu (Champagne, 2010) and with the previous findings of behavioral epigenetics studies with rodents on ELA and the application of these findings to studies with humans (Szyf et al., 2008). From this double perspective, environmental epigeneticists on the MGSS team are particularly interested in ELA as the period in which certain epigenetic traits are thought to stabilize in the brain. Other MGSS researchers, such as those studying clinical and longitudinal cohorts, are interested in additional developmental windows (Geoffroy et al., 2018a,b; Orri et al., 2019) ranging from perinatal influences to adverse social experiences among school-aged children and adolescents that are correlated with neurobiological vulnerability and suicidality.

As Neil Adger (2006) argued in his highly cited article on vulnerability, the concept has been widely used in both the physical and the social sciences, yet in the study of human–environment interactions the concept has a disputed, albeit shared, meaning. Mapping the construct in environmental and hazard studies, the author notes that despite the different formulations of vulnerability (e.g., as experience, outcome, condition, or process), it is often depicted negatively as “susceptibility to be harmed” (Adger, 2006, p. 269) and is based on three main variables: exposure to stress in any given system, its level of sensitivity (degree of affectation), and its adaptive capacity. Similarly, scholars in epidemiology have defined vulnerability as “a dynamic process of negative adaptation in the face of adversity that is shaped by prior embodiment of extrinsic factors as well as intrinsic characteristics” (Kuh et al., 2003, p. 780). This process is therefore considered to be the opposite of resilience when the latter is defined as involving behavioral and emotional adaptations to similarly adverse and stressful conditions (Kuh et al., 2003). Other scholars in global public health have proposed an integrative model in which vulnerability is seen as both “a condition and a process – a condition of heightened fragility of a population or specific group, and a process that is potentially reversible or avoidable through appropriate interventions” (Zarowsky et al., 2013, p. 5)—a model that reflects Canguilhem's views of about the therapeutic impetus of medicine and the possibility of being “restored to health” as alluded to in the opening quote to this article.

In order to understand the ways in which neurobiological vulnerability to suicide risk is localized, measured, profiled, and, ultimately, modeled in epigenetics research one must first understand how this research engages in a particular way of thinking about the normal and the pathological in terms of risk and about adverse experiences as being the loci of vulnerability and susceptibility to risk. This understanding is particularly relevant to grasping suicide risk and childhood abuse as “members of a constellation of ‘social problems”’ (Hacking, 1991, p. 287) that have been articulated in terms of normality and pathology. In Durkheim (2002) classic study on suicide, for instance, concepts of normalcy and of a state of anomy were used to describe, respectively, the normal physiological and pathological states of social systems, with the latter seen as a specific factor in elevated suicide rates. Durkheim's thesis relied on a statistical view of health as normalcy (i.e., a normal distribution of frequencies) and on a metonymic understanding of the human body and the social system as if these were “organisms” of similar kind and scale (Filipe, 2015). This view speaks directly to what anthropologist Jocelyn Lim Chua (2012, p. 207) described as a particular way of thinking about individual death by suicide being a metonym for collective social suffering, which produces “circularity between different scalar phenomena” ranging from molecular and neurobiological phenomena to familial and social levels of analysis (see also Lloyd and Larivée, 2021b). In this form of scientific reasoning, an event (or measure) is used to stand in for the whole (or aggregate) and often with recourse to binaries (de Sousa Santos, 2018) such as, in the field of biosocial science and epidemiology, individual/population, organism/environment, or intrinsic/extrinsic factors.

It is through these very metonymic and statistical forms of reasoning, as developed in modern medicine, sociology, and epidemiology, that the concept of health became equated with the “normal” while deviance from the norm(al) became the functional equivalent of a pathological state and, thus, a topic of major interest for sociological theory (Hacking, 1991; Rose, 2005). In his famous thesis on the normal and the pathological, the philosopher Georges Canguilhem (1991[1978]) argued that the concept of the “normal” encapsulates notions of both statistical frequency/normalcy and of social norms (i.e., normativity), which enable the regulation of conduct and the sanctioning of deviant behavior (Filipe, 2015). Among social beings, then, health is seen as less of a state of “normalcy” and more of an activity that is deeply normative (Rabinow, 1994; Rose, 1998). These considerations are particularly useful in thinking about health not as a normal physiological state but rather as a normative capacity of modulating and adjusting oneself to one's living environment. It follows that under variable conditions, adaptation in the form of “deviation” from the norm may constitute an adequate response to the contingencies of the milieu and, one might add here, to life's adversities.

The adverse circumstances mapped out in the MGSS research echo in many respects those described by Canguilhem in the lectures he gave during 1946 and 1947:

The relation between the living and the milieu establishes itself as a debate to which the living brings its own proper norms of appreciating situations, both dominating the milieu and accommodating itself to it. This relation does not essentially consist (as one might think) in a struggle, in an opposition. That applies to the pathological state. (.) A healthy life, a life confident in its existence, in its values, is a life of flexion, suppleness, almost softness.” (Canguilhem, 2008[1952], p. 113)

As compared to this “healthy life,” which is characterized by the ongoing processes of adapting to and affecting the environment, the life trajectories tracked by the MGSS might be seen as those of a pathological state in which an organism struggles against its milieu (Canguilhem, 2008[1952], p. 113). Similar to the experiences described by the MGSS in the form of early adversity, repeated states of “organic alarm” are associated, in Canguilhem's philosophy, with adverse environments that lead to the normalization of “disordered tension” (Canguilhem, 1978, p. 3). Yet “these normal (that is biologically favorable) reactions end up wearing out the organism in the case of abnormal (that is statistically frequent) repetitions of situations which generate the alarm reaction. In certain individuals, dis-adaptation diseases are set up.” (Canguilhem, 1978, p. 3–4). Disease can be defined, in this very sense, as a reduced “margin of tolerance for the environment's inconstancies” (Canguilhem, 1991[1978], p. 199 in Pentecost and Meloni, 2020). Vulnerability, when compared to the “suppleness” of life (Canguilhem, 2008[1952], p. 113) in which the person is seen as adaptive toward the environment, implies the erosion of one's ability to adapt to the inhabited environment, particularly in situations of extreme variability or in persistently adverse milieux.

In the case of environmental epigenetics, however, such relations hinge on the embodiment of adverse early environments in the form of molecular tags and their hypothesized behavioral expressions and responses (Turecki, 2016; Bouvette-Turcot et al., 2018) over the lifespan. It is hypothesized in the epigenetics literature that these traits and responses, which are thought to help survive stressful circumstances in childhood and adolescence, may become disadvantageous in other situations later in life. The result of this hypothetical mismatch between embodied exposures to adversity and different living milieux, it is believed, is over-reactivity to stressful situations later in life (Barnett-Burns et al., 2018b). The MGSS researchers depict this process as an adversity-based, circular form of neurobiological vulnerability, in which measurable, epigenetic traits^4^ are seen to be impressed on the plastic brain and, by metonymic inference, embedded on the developing self, thereby setting the person on trajectory of emotional reactivity, psychiatric conditions, and suicide risk behaviors. In the words of MGSS director, Gustavo Turecki, early adversity and childhood abuse “lead to the development of maladaptive trajectories… So, suicide risk is, perhaps the most severe negative end point of those psychopathological conditions that are, in turn, predicted by these negative trajectories” (see also Lloyd and Larivée, 2021a). In these models, adverse experiences are identified, then, as key factors for the development of psychopathological conditions and, thus, as predictors of maladaptive trajectories and increased susceptibility to the development of psychiatric conditions and suicide risk.

### Vulnerability and Risk-Thinking in Epigenetics and Mental Health Research

Researchers in the MGSS who study neurobiological traits measure ELA *retrospectively* through psychological autopsies (i.e., post-mortem clinical interviews with people who were close to the deceased). The biological evidence for these specific neuromolecular and epigenetic traits associated with adverse experiences is sought in post-mortem studies of human brain tissue (see Barnett-Burns et al., 2018a,b). These embodied traits are thought to result in a risk profile or trajectory that is characterized by neurobiological vulnerability and maladaptation to stress, which are reflected in overreactions to stress in various living environments later in life. These risk profiles and trajectories may be characterized, in turn, by disadvantageous decision-making and a host of negative social and mental health outcomes (Jollant et al., 2011). While early MGSS epigenetics studies focused on childhood abuse and on impulsive-aggressive behaviors as moderators of psychopathology and suicide risk (McGowan et al., 2009; Lutz et al., 2017), more recent studies also include peripheral sampling (i.e., using blood samples) and the study of epigenetic changes resulting from psychopharmacological treatments with antidepressants (Fiori et al., 2017; Belzeaux et al., 2019). Alongside these studies, longitudinal experimental research has been conducted *prospectively*, in which psychiatric epidemiology is analyzed in parallel with clinical studies involving young people who are deemed to be at risk of suicide. The aim of this research is to investigate socio-environmental and familial indicators as well as peer victimization (e.g., bullying and cyber-bullying) as factors in the development of risk behaviors and negative trajectories (Geoffroy et al., 2016; Perret et al., 2020).

In neuropsychiatry and mental health research, however, models of vulnerability and risk profiles take on added layers of meaning that are of social, ethical, and scientific significance. Indeed, the neuroscientific conception of “vulnerability” is premised on a longstanding way of thinking about human conduct and mental health in terms of risk. As sociologist Nikolas Rose (2005, 2010) has argued, neuropsychiatry has brought together two different images of risk. One that pictures risk as a continuum, along which any individual could be placed, and another image that relies on a categorical and dichotomist view of normal conduct and pathological behavior, which includes the behaviors and profiles of individuals who are identified as being at risk of harming themselves or posing such risk to others. Contemporary psychiatry and neuroscience have, therefore, held the promise of better identifying those “*individuals at risk* – whose particular combination of biology and life history makes *them themselves s*usceptible to some future condition [or any form of] psychiatric disorder” (Rose, 2010, p. 80, original emphasis). This particular model of vulnerability and risk susceptibility concurs, according to Rose (2010), with three main trends in mental health and neuroscientific research: (1) the use of the neurobiological lens to understand and localize normal and pathological conduct, (2) the development of new instruments of risk assessment that identify individual susceptibility, and (3) the impetus to intervene in order to restore health, as alluded to in the opening quote from Canguilhem to this article, or to preempt risk, thereby, preventing potentially negative outcomes.

This framing of psychiatric risk, which has been actualized by the neuromolecular and biosocial turns in post-genomic science (Abi-Rached and Rose, 2010; Meloni et al., 2017), has become even more salient in the development of risk instruments, such as the adverse childhood experiences (ACE) scales, and early intervention programs targeting the early living milieu, including family environments and intimate relations (Gillies et al., 2016; White et al., 2019). As highlighted in a recent overview of the epigenetics literature, early life adversity is a “major risk factor for multiple negative health outcomes later in life, including psychopathology, and therefore represents a significant public health concern” (Barnett-Burns et al., 2018b, p. 116). Due to an overall research focus on risk factors and negative variables and outcomes, however, ELA-based vulnerability is portrayed negatively as a precursor of psychiatric conditions and suicide risk behaviors, as well as a marker (or predictor) of a maladaptive life trajectory. As such, the overall MGSS research provides the tools for thinking about mental ill-health in terms of a new normative risk framing that is punctuated, on the one end, by images of plastic brains and, on the other end, by profiles of embedded neurobiological vulnerability to suicide risk—the worst possible outcome and the extreme end point of an early life struggle with/in an adverse milieu.

Correlating specific life experiences with profiles of psychiatric illness and suicide risk via epigenetic marks presents, however, with the challenge of effectively distinguishing between a signal that is thought to be associated with the embedding of an adverse milieu in early life and a signal that might indicate a molecular “noise” of a lifetime of experiences and other embodied milieux. Early MGSS research on the epigenetics of suicide tended to restrict its attention to the effects of extreme, negative early life experiences whose effects have already been established in research on model organisms. However, as we explore next, the overall MGSS research, which has encompassed multiple axes of clinical, neuroanatomical, and epidemiological research on the cumulative effects of life experiences (Lloyd et al., 2020), suggests that people who have died by suicide often experienced stress and adversity throughout their lives. This research could place a wider range of events and adversities, including those experienced in family environments and in social contexts during adolescence and adulthood, on similar etiological footing. Despite this, the extent to which environmental epigenetics research at the MGSS can contribute to more dynamic portraits of people's lives and deaths remains limited. As researchers are increasingly able to reliably integrate information gleaned from peripheral biomarkers into understandings of neurobiological processes—in ways that might bring them more directly into conversation with findings from other clinical, developmental, and epidemiological research at the MGSS—visions of the complex effects of adverse experiences on mental health and vulnerability might change in years to come.

## Modeling Early Adversity as a Major Risk Factor

Studies of ACEs are a part of an increasingly substantial and influential body of research on the role of early adversity on health and behavior, which emerged in the 1990s (Felitti et al., 1998) and was further developed in following decades (for a critical review, see Edwards et al., 2019). These models of early adversity and its hypothesized biological consequences (in the form of embodied epigenetic traits) enable MGSS researchers to envision a critical link between ELA, defined as a major risk factor (Barnett-Burns et al., 2018a,b), and the development of mental health conditions and risk behaviors later in life, such as depression, personality disorders, and suicidality (Turecki and Brent, 2016)^4^. Researchers in the MGSS argue that epigenetic traits correlated with ELA provide the molecular basis for psychiatric conditions and suicide risks, while also acknowledging that people may respond very differently to those negative early life events (McGirr and Turecki, 2007).

The research of the MGSS has been conducted *retrospectively*, for the most part. This research relies on limited entry points into the living milieu of the person who died by suicide and on specific assessment tools and scales, such as proxy-based clinical interviews (e.g., family member respondents) and standardized life trajectory interviews (Séguin et al., 2011, 2014). Among these tools, psychological autopsies provide the greatest insights into what is believed to be the most potent milieu—early childhood—in terms of shaping a person's subsequent life and neurobiological vulnerability. Among these assessment tools is the Childhood Experience of Care and Abuse Interview (or CECA), which epitomizes the MGSS research interests in ELA. The CECA is based on four principal measures (i.e., indifference/neglect, family tension and conflicts, physical abuse, and sexual abuse) that assess the scale and kind of the adversity and abuse that were experienced (e.g., such as being hit repeatedly). The CECA assessment is based on reports done by the next of kin (Bifulco et al., 1994) and on a combination of categorical and non-categorical questions about the nature and frequency of the abuse (Bifulco et al., 2005). These reports are, first, scored and ranked by severity providing what is seen as a “factual” measure (and scale) of experiences of abuse and neglect^5^. The resulting scores have enabled the development of dichotomy-based typologies of suicide (such as suicide with and without abuse or with and without depression, Lutz et al., 2017), that inform their epigenetic models of neurobiological vulnerability and the retrospective profiling of people who die by suicide. Research at the MGSS has identified differences in multiple epigenetic traits (post-mortem) when comparing the brains of deceased people who have experienced ELA with those who have not (Lutz et al., 2020). This retrospective epigenetics research portrays suicide later in life as the result of the imprint of early negative milieus, with intervening life experiences and precipitating events eclipsed in terms of their etiological weight (Lloyd and Larivée, 2021a).

Running parallel to this retrospective research are developmental studies at the MGSS that draw on *prospective* longitudinal and experimental designs, including research with birth cohorts and psychiatric epidemiological studies in suicidality (Geoffroy et al., 2016; Orri et al., 2019). Their main aim is to map particular types of adversity in childhood and adolescence (such as peer victimization and bullying) that can predict suicide risk later in life, alongside cumulative and interactive risk variables (such as the chronicity, severity, and context of experiences of abuse) seen as potential moderators of psychiatric conditions and suicide risk behaviors. As a whole, these prospective studies broaden attention to life's experiences and risk trajectories by considering the potential impacts of other living milieus on school-aged children and adolescents (Séguin et al., 2011, 2014), including those experienced in public social spaces. Researchers have characterized this broader outlook as a shift from “variable-oriented” to “person-oriented” research (Séguin et al., 2014, p. 124) on life trajectories that affords a more granular understanding of “conditional and cumulative probabilities and contextual factors” of risk (Séguin et al., 2014, p. 125). In order to develop a portrait of these biosocial interactions and cumulative effects, the psychiatric epidemiology research conducted at the MGSS considers a whole range of risk factors that move young people onto trajectories of neurobiological vulnerability and suicidality (Geoffroy et al., 2018a,b; Orri et al., 2018; Perret et al., 2020). In this purview, MGSS researchers have devised “multi-trajectory models” that trace the longitudinal-developmental course of suicide risk in relation to personality traits (e.g., childhood irritability) and mood disorders (Orri et al., 2018, p. 469).

Alongside this research axis are studies of longitudinal birth cohorts that focus on a wider range of suicide risk factors, ranging from those identified in early and perinatal life (e.g., low birth weight, Geoffroy et al., 2014) to ongoing (or more recent) experiences of peer victimization and cyberbullying (Geoffroy et al., 2016; Perret et al., 2020). One of the birth cohort studies that they have analyzed is the Québec-based QLSCD (Québec Longitudinal Study of Child Development), a longitudinal study of 2120 people born in 1997–1998 with the motto “I am, I'll be: The survey on the future of a generation.” On the basis of this study and the comparison of three trajectories of peer-victimization—low, moderate, and severe from the ages of 6–13 years old—MGSS researchers report that adolescents who were more severely victimized throughout their school journey are “at greater chance of suicidality in adolescence than less severely victimized children, even accounting for a plethora of confounders assessed throughout childhood” (Geoffroy et al., 2018a, p. 41). Another cohort study of interest to the team is the 1958 National Child Development Study (NCDS) based in Britain, in which these researchers further identified perinatal risk factors (e.g., young maternal age), and developmental risk factors (i.e., externalizing behaviors) and correlated these with the number of childhood adversities that were associated with suicidality later in life (Geoffroy et al., 2018b). The findings produced through these cohort study analyses, thus far, are consistent with those from previous ACEs studies (Anda et al., 2006), in which researchers identified “a dose-response association between the number of emotional adversities and suicide, with the highest suicide risks [found] among those experiencing three or more adverse experiences” (Geoffroy et al., 2014, p. 10).

The portrait of vulnerability to suicide risk that emerges from these studies places the living milieu of ELA at the core of the environmental epigenetics research of the MGSS but considers it to be a risk factor that must be tallied in a broader assessment of vulnerability to suicide, which includes “hazard ratios” (Geoffroy et al., 2014, p. 1250) as well as gradients and co-factors of risk. As a result, while similar factors are salient in the development of suicide risk and neurobiological vulnerability models across cohort and epigenetics research, in cohort research those factors are specifically described in terms of an attributable risk fraction at the population level, that is, the proportion of suicides (as calculated by the researchers) that would have been prevented if those risk factors had not been present (Geoffroy et al., 2014). This hypothesis helps tempering the Québec cohort study motto (Lloyd et al., 2020), shifting it from being a statement of fact—“I am, I'll be”—to a potential condition—“I am, I might be(come)”—which alludes to a more indeterminant process of being and becoming (Biehl and Locke, 2017).

Thus, on the one hand, environmental epigenetics frames neurobiological vulnerability and being at-risk of suicide as correlated to a specific set of essential risk factors and adverse experiences in early life, such as childhood abuse. In cohort studies, on the other hand, these conditions remain the key risk factors for the onset of a trajectory that is marked by neurobiological vulnerability (in the form of suicidality), although it is thought that this vulnerability might be modulated by additional adverse experiences (e.g., bullying) later in life and by contextual factors in developmental research. Both research axes speak to the embedding of vulnerability, but environmental epigenetics studies tend to focus on essential risk factors and how trajectories of adversity and maladaptation can lead to psychiatric conditions and suicidality, whereas cohort and developmental studies consider how constellations of risk factors might be associated with differential pathways of vulnerability.

### The Adverse Milieu as the Locus of Risk and Vulnerability

While elsewhere we have drawn attention to temporal aspects of risk assessment in retrospective and prospective research (Lloyd et al., 2020), here we would like to consider the ways in which different adverse milieus are thought to produce vulnerability. The powerfully formative experience of adversity in early life is thought to set a person on a trajectory of elevated neurobiological vulnerability to suicide risk and mental ill health. The risk of future sickness, by virtue of the embodied effects of adversity, has left the person “diminished” (Canguilhem, 1978) in the sense that their acquired epigenetic traits are considered to make them more likely to overreact (or to react adversely) to future negative experiences (Lloyd and Larivée, 2021a). Vulnerability means, in this context, that their equanimity and adaptiveness has been reduced while their sensitivity to stress and susceptibility to harm has increased. Thus, one of the hypotheses posited in the epigenetics literature is that experiences of adversity during windows of increased sensitivity and plasticity have “a potent, yet complex, impact on the developmental programming of the stress response” (Barnett-Burns et al., 2018b, p. 116) that may engender an eventual “mismatch” between developmental cues/alterations and later-life environments and experiences (for details, see Barnett-Burns et al., 2018b, p. 125). This hypothetical mismatch would result in a situation similar to what Canguilhem (1978, p. 3) described as “dis-adaptation diseases” and organic “alarm responses” to future adverse and stressful situations. In epigenetics models, the localization of these diseases and responses lays, however, at the level of early-life exposures and (mal)adaptive reactions that become biologically embedded and then reenacted at the level of neurobehavioral traits and responses.

Can this lost trait of equanimity be “restored”? Can embodied forms of adversity and vulnerability “leave” (Canguilhem, 1978)? To the time of writing, studies conducted by the MGSS have no definitive answer to these questions, which are of central importance to understanding the vision of vulnerability that is portrayed in their environmental epigenetics research. Given that epigenetic traits are tissue specific (i.e., if one wants to know what is happening in the brain, one needs to sample the brain) and despite the fact that there are increasingly strong correlations identified between peripheral epigenetic changes and brain-based changes, reliable and robust correlations remain elusive. In other words, while findings from the early epigenetics studies suggest that epigenetics traits acquired in the early years can be altered in the adult brains of rodents (Weaver et al., 2005), among humans the scientific hypothesis of “reversibility” remains a hypothesis. Researchers at the MGSS are currently tackling one angle of this question through studies of the potential mitigating effects of therapeutic interventions, specifically in studies on the role of antidepressant treatments in differential methylation patterns and their role as predictive biomarkers (e.g., Belzeaux et al., 2019). However, the results that are emerging from this research do not advance causal models of reversibility such as those documented in epigenetics studies with rodents (c.f., Fiori et al., 2017; Fiori and Turecki, 2020). Similarly, evidence for the association of mild stress exposures with “increase[d] resilience to future stressors” (Barnett-Burns et al., 2018b, p. 124) is limited and has been gleaned from those same studies with animal organisms.

In the interim, other forms of evidence, such as that derived from research with different cohorts, provide different insights into the lives of people who experienced adversity that might come to be described and enrolled in future epigenetics research on ELA (Barnett-Burns et al., 2018a,b). There are at least two possible scenarios that might emerge from this tentative future research. First, molecular researchers may find that some people who experience ELA do not acquire the epigenetic traits associated with profiles of mental illness and suicide. Second, researchers may identify people who acquire epigenetic traits that are correlated with mental distress and vulnerability to suicide risk as well as with greater reactivity and heightened stress responses to later life experiences. Attention to later life experiences, in more dynamic models and research agendas, could provide insights into why people would remain on (or would potentially shift away from) particular life trajectories. Although Canguilhem believed that the potential exposures to and “excitations” arising from the environment were infinite, he also foresaw that the ways in which a person can be oriented toward—and even “debate” with—the environment are limited. He therefore concluded (Canguilhem, 2008[1952], p. 113) that “an organism is thus never equal to the theoretical totality of its possibilities” (Canguilhem, 2008[1952], p. 120).

Future epigenetics studies based on more dynamic models may come to suggest that epigenetics traits associated with embodied forms of vulnerability persist in some cases but may also demonstrate that the embedding of vulnerability may shift over time and be contingent on one's living milieus and their various potentials (Taussig et al., 2013). These dynamic and integrative studies would have to consider, then, a multitude of adverse milieus and life experiences alongside variable developmental pathways, individual profiles, and social environmental contingencies, which reflect a consideration of how the person lives and inhabits the environment. Consequently, the epigenetic landscape of embodied adversity would have to be calibrated through further investigation on the embedding of vulnerability and its dynamic interplay with present and/or future living milieux beyond correlative models of psychiatric and suicide risk. The possibility remains, therefore, that future experimental research might be construed and operationalized in a way that may explore how different relationships are established (or might be established) between the living and the milieu, as envisioned in Canguilhem's philosophy. By looking at the formative potency of early adverse experiences alongside the effects of subsequent living milieus, the MGSS research supports a biosocial explanatory framework for neurobiological vulnerability and, more broadly, a new scientific framing of the risk of mental distress and suicide, which carry significant conceptual, societal, and translational implications.

## Discussion: is Embedded Vulnerability a “New Pathological?”

What is striking about the MGSS research is not its exceptionality among models of neurobiological risk, but its broader alignment with contemporary post-genomic and biosocial understandings of early adversity, plasticity, and vulnerability (Meloni, 2014, 2019; Lappé, 2018). As these understandings of the human body and the brain have become instrumental to current global and public health research agendas, such as DOHaDs, early life and the adverse milieu have taken center stage as a core analytic that can be used to understand people's lives and health trajectories (Lappé and Landecker, 2015; Pentecost and Meloni, 2020). When specific findings from groups like the MGSS converge with these broader agendas, however, the strength of their studies—that are grounded in specific risk instruments and variables that seek to reliably track adverse experiences and trajectories of vulnerability—does not always translate seamlessly. Indeed, voices from across the social sciences, bioethics, and critical public health have sought to unpack and critically engage with the concept of vulnerability. While some have highlighted the ambiguity and vagueness of existing definitions in broader research literature (Katz et al., 2019), others have drawn attention to its conceptual limitations and contextual heterogeneity, particularly when definitions of what constitutes a “vulnerable” group that exclude people's own definitions and experiences (Zarowsky et al., 2013; Benmarhnia et al., 2018; McLaren et al., 2020).

In environmental epigenetics research, vulnerability and adaptation are only ever considered in terms of what are thought to be situated, specific mechanistic reactions to the milieu at a given moment. In the environmental epigenetics research conducted at the MGSS, the specific contexts to which their models have applied more regularly are childhood abuse and extreme adversity as measured by the CECA. Thus, there is general agreement that adaptation and vulnerability are contingent on particular environmental and socio-temporal conditions. Yet the signposts of life that have emerged from the earlier MGSS research are being rapidly generalized into clinical and developmental studies as well as broader understandings of health and life trajectories, through comparative analyses of these trajectories and correlations of risk variables that are tracked in birth cohorts. As a result, the model of neurobiological vulnerability that emerges from prospective cohort research seems to imply a decontextualized “universality” of biosocial variables and life trajectories, as these materialize in specific geographic and socio-historical milieus and yet are mobilized (and correlated) across very different contexts and datasets. While this might be a hallmark of metonymic and statistical reasoning, it is not without theoretical and methodological implications, particularly in environmentally informed research that focuses on the effects of specific, context-dependent life experiences. It is worth noting, as Canguilhem (2008[1952], p. 118–120) wrote, that one of the “functions of science is to devalorize the qualities of objects that comprise the milieu proper to the [hu]man [so as to form a] general theory of a real, that is to say, inhuman milieu. (.) And thus the milieu proper to [the hu]man is not situated within the universal milieu [of science] as contents in a container.”

Alongside these questions, there are also concerns regarding the clinical, ethical, and societal implications of epigenetic knowledge translation. For instance, in the epigenetics literature it was proposed that the effectiveness of interventions and treatments of exposure to adversity would depend on tailoring them to susceptibility factors at the individual level (Barnett-Burns et al., 2018b). Notwithstanding, the existing social science and ethics literature suggests that the use of biomarkers in neuropsychiatric research and in the development of diagnostic and therapeutic tools is problematic (Singh and Rose, 2009). This is due to the variability and high specificity of those eventual markers (that, in the case of epigenetics research, are also tissue-, variable-, and/or person-specific) and hence potentially misleading and/or lacking predictive value when applied to broader segments of the population. Similarly, others have argued that population health data has low predictive value when it is applied to developing and transitioning subgroups of people, such as children and adolescents (Sawyer et al., 2018), for whom presumed health trajectories and risk probabilities might not come to pass. Equally, as scientific portraits of vulnerability and risk become ingrained in public health and social policy literature, there are concerns about the ways in which they may come to inform early interventions that are based on risk assessments and childhood adversity measurements (Edwards et al., 2019), as well as public health interventions and prevention strategies based on neurobiological and biosocial understandings of socio-economic deprivation (Pitts-Taylor, 2019). In the specific context of suicide risk, mental health and suicide prevention programs may stumble on similar and longstanding critiques of risk reduction and prevention strategies, that have been depicted as either overly individualized or as too decontextualized and apolitical (White et al., 2015).

Paradoxically, then, by arguing for a novel understanding of the human body and brain, in which life “debates” with the milieu, as Canguilhem described it, epigenetics redefines vulnerability as an embedded neuromolecular condition that results from the embodiment of an adverse milieu and a reverberating pathological state that has the potential to be fixed (Pitts-Taylor, 2019). “Fixed” in this context has the double sense of being stabilized in one's life trajectory yet potentially also resolved or restored, as alluded to in our opening quote. In a risk-suffused milieu such as that of ELA, the “fixing” of vulnerability implies the identification of those who are considered “at-risk” as well as early interventions in adverse (and presumably “unhealthy”) living milieux. In effect, developmental and epigenetics research have provided increasingly stronger evidence that those who are more sensitive to the environment and negative exposures are also those who are more responsive to positive experiences and interventions (Barnett-Burns et al., 2018b). Yet a word of caution must be voiced if we are to take this proposition and Canguilhem's philosophy of life seriously. Any intervention that, in seeking to “fix” the damage caused by a negative exposure, would imply the taming of risk or a radical change of living milieu, may imply, in turn, a new set of formative events and interactive effects that could be experienced, paradoxically, as an adversity or as a loss of agency and thereby generate unexpected forms of “risk.” These generative effects would also include the development of negative risk subjectivities (Singh and Rose, 2009; Rose, 2010; Gillies et al., 2016) and social stigma, both experienced and enacted (Gray, 2002), because specific living environments and already marginalized groups may be problematically labeled as “vulnerable” (Zarowsky et al., 2013) and, in turn, targeted for precautionary interventions and risk prevention strategies. Yet, as recent empirical studies have also shown, biosocial understandings of risk and, increasingly also, resilience may be used in ways that are “neither inherently liberatory nor inherently oppressive” (Müller and Kenney, 2020, p. 2) but instead are choreographed with narratives of restorative justice and trauma-informed care for their political effects (e.g., achieving institutional reforms in the juvenile correction system).

Our particular concern is that if environmental epigenetics research is enrolled in early interventions whose end goal is to “restore” what may have been “lost,” the vision of life produced in the field will remain mechanistic and framed in terms of “bad”/“good” epigenetic traits, healthy and unhealthy brains and life trajectories, and positive and negative adaptations, along a particular scale of risk. This has two foreseeable consequences. First, overcoming neurobiological vulnerability might be seen as the natural goal of therapeutic interventions. In this process, some dimensions of vulnerability, such as plasticity and sensitivity, might remain understudied or undervalued, whereas others may be targeted for risk mitigation and prevention strategies. Second, the possibility of overcoming vulnerability may come to the fore as part of a new biosocial equation of pathology vis-à-vis normality that is articulated in terms of risk and maladaptive life trajectories that are rooted in ELA. In this purview, adaptation, early intervention, and resilience building might come to be seen as something to be valued and cultivated in everyday life and in public policy as a way of preventing, or at least mitigating, the negative potential of adversity in risk-suffused environments. Much like health and deviancy became synonyms for the “normal” and the “pathological” in the modern medical and social sciences, vulnerability may stand out as a negative condition in post-genomic biosocial science that forebode a “new pathological,” while resilience might gradually stand in as a positive adaptation to the environment that points toward a “new normal” way of living in and coping with stressful and persistently adverse milieux.

## Conclusion: From Biosocial Science to an Ecosocial Framing of Vulnerability and the Living Milieu

We began this article by showing how epigenetic science is anchored in a conceptually novel, albeit persistent, way of thinking about neurobiological vulnerability and mental health-related risk in terms of normalcy/pathology. Given the focus of the MGSS epigenetics research on risk factors and severe stressors, neurobiological vulnerability has been more often portrayed negatively as a precursor of maladaptive trajectories and a predictor of neuropsychiatric risk. As we showed next, these risk factors can be localized in the epigenetic landscapes of post-mortem brain tissue and correlated to experiences of adversity that are assessed by proxy, through clinical interviews and questionnaires. The experience of ELA is, in turn, examined in developmental and longitudinal studies in terms of how it interacts with other commensurable life experiences (e.g., bullying) and behavioral traits (e.g., irritability) in childhood and youth. Overall, the profile of the vulnerable person who is at risk of developing a psychiatric condition and suicide risk behaviors may be portrayed as that of someone on a maladaptive life trajectory whose extreme end point (i.e., suicide), while not inevitable, is associated with an exposure to adversity, abuse, neglect, and victimization. Current methodological limitations in environmental epigenetics that are related to the tissue specificity of epigenetic traits mean that, at the moment, researchers cannot produce molecular evidence that would support more dynamic portraits of people's lives and deaths, such as those that may emerge from epidemiological and clinical research. To put it differently, even though the possibility remains that people might stay on or shift away from a maladaptive trajectory, a more mechanistic model of neurobiological vulnerability persists in this field: exposure to ELA is correlated with a higher susceptibility to harm, mental distress, and suicide risk, whether this exposure is construed as formative or cumulative.

Hence, existing technoscientific repertoires of risk anticipation and prediction (Adams et al., 2009; Rose, 2010), which bring the potential future to bear on the present living environment, are overlaid, in epigenetics and biosocial research, with new models and measurements of “past” risk factors that are actualized through an embodied and reverberating adverse milieu. In these models of psychiatric risk, little room seems to be left for situated analyses of mental distress and suicidality that are linked, for example, to socio-political injustice as experienced by particular communities and groups of people (Stevenson, 2014). In this article, we also showed that the combination of retrospective epigenetics studies and prospective epidemiological orientations in these models may reinforce, paradoxically, an arid biosocial framing of ELA-based neurobiological vulnerability as an epigenetically embedded condition, wherein it is difficult to account for relational dimensions of vulnerability as well as the social ecologies of mental health. Thus, while this combination of research foci makes epigenetics a promising field of research that is directly relevant to public and mental health, translational efforts may be accompanied by problematic applications and decontextualized interpretations of emerging findings that render them in new normative and epistemically “ostentatious” terms, as Martyn Pickersgill (2017, p. 187) put it. When considering the translation of emerging findings into global public health frameworks, early interventions, and risk/harm reduction strategies for improving life trajectories and mental health outcomes, it is worth heeding the challenges and limitations of epigenetics, as noted by scientists themselves (Barnett-Burns et al., 2018a).

To paraphrase Georges Canguilhem (2008[1952], p. 113), the definition of “a healthy life” is a life “confident in its existence, in its [own] values” that is shaped by a relationship of “debate” with the milieu. This definition calls for attention to be paid to the milieu that is “proper” to human beings (Canguilhem, 2008[1952], p. 120), that is, their everyday living environments and social realities. It follows that the specific conditions on which the intertwining of human organism and living environment is scientifically modeled matter for how we understand and order their relationships in terms of healthy/unhealthy ways of living or of normal/pathological forms of relating to and coping with an adverse milieu. From this perspective, we argue that vulnerability may be conceptualized as a both a situated condition and a contingent process (as opposed to a biosocially universal “state,” Zarowsky et al., 2013; Benmarhnia et al., 2018) that materializes in and is actualized by one's living milieu. As such, vulnerability may be seen as more than a “unhealthy state” of acquired susceptibility to risk and harm and heightened frailty; it also encompasses a sense of “genuine plasticity” and sensitivity (Taussig et al., 2013, p. s4) to one's inhabited environment, hence, a relational mode of being vulnerable to others. It is worth considering, therefore, (i) how the relational, social, and political ecologies of vulnerability play out in everyday life and (ii) whether the intertwining of the living and the milieu might be articulated not only in terms of its negative potential (i.e., risk, harm, exposure to stress) but also in terms of positive or transformative potentials, including processes of adaptation and socio-environmental affordances, as well as different forms of healing and repair (Kirmayer, 2019; Rose et al., 2021). We wonder, then, in what ways biosocial research and ecosocial theory might be bridged in a lively, processual perspective that “embraces a social production of disease perspective while aiming to bring in a comparably rich biological and ecological analysis,” as proposed by Nancy Krieger (2001, p. 672).

Through this bridge and by troubling existing models of “neurobiological vulnerability,” we consider how an ecosocial approach to vulnerability might help illuminate three interrelated elements of mental health and distress. First, the pathways of embodiment and the cumulative interplay between exposure, susceptibility, and resistance (Krieger, 2001). Second, the relationship between these pathways and issues concerning moral agency, health equity, and social justice in ways that shift accountability and responsibility from the individual/group levels to the macro-systemic and public policy levels (Krieger, 2012; Filipe et al., 2020). Third, the living conditions of the “person-in-environment” seen as “embedded in an ecosocial crucible of interacting processes” (Filipe et al., 2020, p. 9) that warrant a combination of epigenetic, neurodevelopmental, longitudinal, socio-historical, and ethical empirical perspectives. Thus, alongside the philosophical unpacking of the vitality and normativity of human health and life as advanced by Georges Canguilhem, we have suggested that additional social scientific and empirical research attention should be paid to the people's everyday living conditions and to the social ecologies of adversity, precariousness, vulnerability, and marginalization (Han, 2018; Rose et al., 2021) as these affect people's health and their “life as such” (Fassin, 2009, p. 45; Filipe, 2014).

To this end, critical engagements between the life sciences and the social sciences alongside community and policy engagement might help devising more generative forms of knowledge co-production (Filipe et al., 2017) and empirically situated “research interventions” and methodologies (for a review, see Filipe, 2017; Roberts and Sanz, 2018) that help ground biosocial research in lived realities and experiences. Similarly, even though the dynamics of risk/resilience and adaptation/vulnerability have been extensively studied in developmental and environmental research and critiqued, therein, due to their conceptual and cultural ambiguity (Ungar, 2011; Grove, 2018), considerably less has been said about them in the field of anthropological and social studies of biomedicine and neuroscience where additional and carefully grounded research is needed in the future. We hope that this article takes a first step toward thinking both critically and more creatively about the epigenetic and biosocial landscapes of psychiatric risk by considering the social ecological dimensions of mental health and vulnerability in the adverse living milieu.

## Data Availability Statement

The original contributions generated for the study are included in the article/supplementary material, further inquiries can be directed to the corresponding author/s.

## Author Contributions

AMF conceived of the initial frame for the paper and led the writing process. All authors contributed to funding acquisition in support of this work. All authors contributed to the article and approved the submitted version.

## Conflict of Interest

The authors declare that the research was conducted in the absence of any commercial or financial relationships that could be construed as a potential conflict of interest.

## Footnotes

^1^The terms lifecourse and lifespan have been used interchangeably and with other similar constructs, such as life cycle (Kuh et al., 2003) and life trajectories. For the purposes of this paper, we have chosen to use the term “lifespan,” whenever it is originally used in the scholarship cited, which is the preferred term in developmental and psychological research as opposed to “lifecourse” which is more often used in sociology and demography at the population level. Lifespan seems to capture the qualities of an expansive timeline of human life and its milieus (including early life and *in-utero* environments). Similarly, we shall use “life trajectories” to designate a shared research interest in life's experiences and risk trajectories at the nexus of developmental, epidemiological, and longitudinal research.

^2^We use the term “early (life) adversity” as an analytic and a construct that encompasses negative, stressful experiences, and environmental “exposures” at the center of environmental epigenetics attention (e.g., stress, neglect, maltreatment). It is worth noting, however, that early adversity represents a vast field of research in the social and health sciences and that the term is fraught with considerable ambiguity at the conceptual, operational, and translational levels (Edwards et al., 2019). The concept has also evolved within the field of environmental epigenetics: from the original focus on early life stress (or ELS) in rodent studies (e.g., Weaver et al., 2004) to childhood maltreatment and early childhood abuse (ECA) in studies with human populations (e.g., McGowan et al., 2009) to the all-encompassing term “early life adversity” (ELA) (Bouvette-Turcot et al., 2018) that aligns with the study of adverse childhood experiences (ACEs) (Felitti et al., 1998).

^3^These epigenetic traits include, for example, the DNA methylation state of the GR (glucocorticoid receptor) gene identified in human brain tissue after death (see McGowan et al., 2009) and a global impairment of the myelin-related transcriptional program associated with a history of childhood abuse (Turecki, 2016; Lutz et al., 2017).

^4^For Durkheim (2002, p. 219), anomy “is a regular and specific factor in suicide on our modern society,” and thus differing from other identified states (e.g., egoistic or altruistic) “in its dependence, not on the way in which individuals are attached to society, but on how it regulates them”.

^5^For molecular researchers at MGSS, “suicide with abuse” includes people who report “non-random major physical and/or sexual abuse” up to age 15. Only cases with the maximum severity ratings of 1 and 2 (“marked” or “moderate” on a four-point scale) are included in the latter (Lutz et al., 2017, p. 1186).
